# Supplementary material for: Heat adaptation of phage T7 under an extended genetic code
Source: Virus Evol. 2021 Dec 1;7(2):veab100. doi: 10.1093/ve/veab100 (PMC8923235; doi:10.1093/ve/veab100)
Supplement: veab100_Supp [file veab100_supp.zip › Sequencing_Analysis.zip!/Sequencing_Analysis/S1/evidence/JC_58.html]

BRESEQ :: Evidence


| New junction evidence | | | | | | | | | | | |
| --- | --- | --- | --- | --- | --- | --- | --- | --- | --- | --- | --- |
|  | | seq id | position | reads (cov) | reads (cov) | score | skew | freq | annotation | gene | product |
| \* | ? | NC\_001604.1 | = 16508 | 1353 (0.320) | 2351 (0.570) | 369/586 | 0.2 | 62.6% | intergenic (–/–) | *–/–* | –/– |
|  | ? | NC\_001604.1 | 16970 = | 1483 (0.360) |  |  |  |  | intergenic (–/–) | *–/–* | –/– |

Only 100 of 2575 total aligned reads displayed.  

`CTGGTGCTCTCATCTGCAAACTGTGGATTATCAAGACCGAAGAGATGCTCGTAGAGAAAGGCTTGAAGCATGGCTGGGATGGGGACTTTGCGTACATGGCATGGGTACATGATGAAATCCAAGTAGGCTGCCGTACCGAAGAGATTGCTCAGGTGGTCATTGAGACCGCACAAGAAGCGATGCGCTGGGTTGGAGACCACTGGAACTTCCGGTGTCTTCTGGATACCGAAGGTAAGATGGGTCCTAATTGGGCGATTTGCCACTGATACAGGAGGCTACTCATGAACGAAAGACACTTAACAGGTGC‑‑‑‑‑‑‑‑‑‑‑‑‑‑‑‑‑‑‑‑‑‑‑‑‑‑‑‑‑‑‑‑‑‑‑‑‑‑‑‑‑‑‑‑‑‑‑‑‑‑‑‑‑‑‑‑‑‑‑‑‑‑‑‑‑‑‑‑‑‑‑‑‑‑‑‑‑‑‑‑‑‑‑‑‑‑‑‑‑‑‑‑‑‑‑‑‑‑‑‑‑‑‑‑‑‑‑‑‑‑‑‑‑‑‑‑‑‑‑‑‑‑‑‑‑‑‑‑‑‑‑‑‑‑‑‑‑‑‑‑‑‑‑‑‑‑‑‑‑‑‑‑‑‑‑‑‑‑‑‑‑‑‑‑‑‑‑‑‑‑‑‑‑‑‑‑‑‑‑‑‑‑‑‑‑‑‑‑‑‑‑‑‑‑‑‑‑‑‑‑‑‑‑‑‑‑‑‑‑‑‑‑‑‑‑‑‑‑‑‑‑‑‑‑‑‑‑‑‑‑‑‑‑‑‑‑‑‑‑‑‑‑‑‑‑‑‑‑‑‑‑‑‑‑‑‑‑‑‑‑‑‑‑‑‑‑‑‑‑‑‑‑‑‑‑‑‑‑‑‑‑‑‑‑‑‑‑‑‑‑‑‑‑‑‑‑‑‑‑‑‑‑  >  NC_001604.1/16202‑16508`  
`‑‑‑‑‑‑‑‑‑‑‑‑‑‑‑‑‑‑‑‑‑‑‑‑‑‑‑‑‑‑‑‑‑‑‑‑‑‑‑‑‑‑‑‑‑‑‑‑‑‑‑‑‑‑‑‑‑‑‑‑‑‑‑‑‑‑‑‑‑‑‑‑‑‑‑‑‑‑‑‑‑‑‑‑‑‑‑‑‑‑‑‑‑‑‑‑‑‑‑‑‑‑‑‑‑‑‑‑‑‑‑‑‑‑‑‑‑‑‑‑‑‑‑‑‑‑‑‑‑‑‑‑‑‑‑‑‑‑‑‑‑‑‑‑‑‑‑‑‑‑‑‑‑‑‑‑‑‑‑‑‑‑‑‑‑‑‑‑‑‑‑‑‑‑‑‑‑‑‑‑‑‑‑‑‑‑‑‑‑‑‑‑‑‑‑‑‑‑‑‑‑‑‑‑‑‑‑‑‑‑‑‑‑‑‑‑‑‑‑‑‑‑‑‑‑‑‑‑‑‑‑‑‑‑‑‑‑‑‑‑‑‑‑‑‑‑‑‑‑‑‑‑‑‑‑‑‑‑‑‑‑‑‑‑‑‑‑‑‑‑‑‑‑‑‑‑‑‑‑‑‑‑‑‑‑‑‑‑‑‑‑‑‑‑‑‑‑‑‑‑caggtgcGATTGTCCCCAATGGTAAACAGAAGGAAATGATTGTCCAGTTCCTGACACACG‑‑GTATGGAAGGATTGATGACATTCGTAGTACGTACATCATTTCGTGAGGCCATTAAGGACATGCACGAAGAGTATGCAGATAAGGACTCTTTCAAACAATCTCCTGCAACAGTACGGGAGGTGTTCTGATGTCTGACTACCTGAAAGTGCTGCAAGCAATCAAAAGTTGCCCTAAGACTTTCCAGTCCAACTATGTACGGAACAATGCGAGCCTCGTAGCGGAGGCCGCTTCCCGTGGTCACATCTCG  >  NC_001604.1/16970‑17269`  

                                                                                                                                                                                                                                                                                                                                                                                                                                                                                                                                                                                                                                   
`ttgtTGCTATCATCTGAAAACTGTGGATTATCAAGACCGAAGGGATGCGTGTAAAGAAATGGTGTAAGCTGGGCTGGGATAGGAACTTTGCGTACATGGCATGGGTACATGATGAAATCCAAGTAAGCGGCCGTACCGAAGAGAATTCTCAGGTGGTCAGTGAGACCGAACAAGAAGCGATGCGCTGGGTTGGAGACCACTTGAACTTCCGGTGTCTTCTGGATAACGAAGGTAAGATGGGTCCTAATTGGGCGATTTGCCACTGATACAGGAGGCTACTCATGAACGAAAGACACTTAAC                                                                                                                                                                                                                                                                                                                      <  1:616272/297‑1`  
   `gacagCTCATCTGCAAACTGTGGATTATCAAGACCGAAGAGATGCTCGTAGAGAAAGGCTTGAAGCATGGCTGGGATGGGGACTTTGCGTACATGGCATGGGTACATGATGAAATCCAAGTAGGCTGCCGTACCGAAGAGATTGCTCAGGTGGTCATTGAGACCGCACAAGAAGCGATGCGCTGGGTTGGAGACCACTGGAACTTCCGGTGTCTTCTTGATACCGAAGGTAAGATGGGTCCTAATTGGGCGATTTGCCACTGATACAGGAGGCTACTCATGAACGAAAGACACTTAACAGG                                                                                                                                                                                                                                                                                                                   <  1:232745/296‑1`  
   `agGCTTTCATCTGCAAACTGTGGATTATCAAGGCCGATGGGATGCTCGTAGACAAAGGCTTGAAGCATGGCTGGGATGGGGCCTTTGCGTACATGGCATGGGTACATGATGAAATCCAAGTAGGCTGCCGTACCGGAGAGATTGCTCAGGTGGTCATTGAGACCGCACAAGTAGCGATGCGCTGGGTTGGAGACCACTTGAACTTCCGGTGTCTTCTGGATACCGAAGGTAAGATGGGTCCTAATTGGGCGATTTGCCACTGATACAGGAGGCTACTCATGAACGAAAGACACTTAACAGG                                                                                                                                                                                                                                                                                                                   <  1:373294/299‑1`  
   `GTGCTCTCATCTGCAAACTGTGGATTATCAAAACAGAAGAGAGGCTCGTAGAGGAAGGCTGGAAGCCTGGCTGGGATGGGGACTTTGGGTACATGGCATGGGGACATGATGAAATCCAAGTAGGCTGCCGTACCGAAGAGATTGCTCAGGTGGTCATGGAGACCGCACAAGAAGCGATGCGCTGGGTTGGAGACAACTGGAACTTCCGGTGTCTTCTGGATACCGAAGGTAAGATGGGTCCTAATTGGGCGATTTGCCACTGATACAGGAGGCTACTCATGAACGAAAGACACTTAACAGG                                                                                                                                                                                                                                                                                                                   <  1:140114/301‑1`  
     `GCTCTCATCTGCAAACTGTGGATTATCAAGACCGAAGAGATGCTCGTAGAGAAAGGCTTGAAGCATGGCTGGGATGGGGACTTTGCGTACATGGCATGGGTACATGATGAAATCCAAGTAGGCTGCCGTACCGAAGAGATTGCTCAGGTGGTCATTGAGACCGCACAAGAAGCGATGCGCTGGGTTGGAGACCACTGGAACTTCCGGTGTCTTCTGGATACCGAAGGTAAGATGGGTCCTAATTGGGCGATTTGCCACTGATACAGGAGGCTACTCATGAACGAAAGACACTTAACAGGTG                                                                                                                                                                                                                                                                                                                 <  1:28552/301‑1`  
          `CATCTGCAAACTGTGGATTATCAAGACCGAAGAGATGCTCGTAGAGAAAGGCTTGAAGCATGGCTGGGATGGGGACTTTGCGTACATGGCATGGGTACATGATGAAATCCAAGTAGGCTGCCGTACCGAAGAGATTGCTCAGGTGGTCATTGAGACCGCACAAGAAGCGATGCGCTGGGTTGGAGACCACAGGAACTTCCGGTGTCTTCTGGATACCGAAGGTAAGATGGGTCCTAATTGGGCGATTTGCCACTGATACAGGAGGCTACTCATGAACGAAAGACACTTAACAGGTGCGATT                                                                                                                                                                                                                                                                                                            <  1:27839/301‑1`  
            `TCTGCAAACTGTGGATTATCAAGACCGAAGAGATGCTCGTAGAGAAAGGCTTGAAGCATGGCTGGGATGGGGACTTTGCGTACATGGCATGGGTACATGATGAAATCCAAGTAGGCTGCCGTACCGAATAGATTGCTCAGGTGGTCATTGAGACCGCACAAGAAGCGATGCGCTGGGTTGGAGACCACTGGAACTTCCGGTGTCTTCTGGATACCGAAGGTAAGATGGGTCCTAATTGGGCGATTTGCCACTGATACAGGAGGCTACTCATGAACGAAAGACACTTAACctgtctcttata                                                                                                                                                                                                                                                                                                          >  1:418729/1‑289`  
            `TCTGCAAACTGTGGATTATCAAGACCGAAGAGATGCTCGTAGAGAAAGGCTTGAAGCATGGCTGGGATGGGGACTTTGCGTACATGGCATGGGTACATGATGAAATCCAAGTAGGCTGCCGTACCGAAGAGATTGCTCAGGTGGTCATTGAGACCGCACAAGAAGCGATGCGCTGGGTTGGAGACCACTGGAACTTCCGGTGTCTTCTGGATACCGAAGGTAAGATGGGTCCTAATTGGGCGATTTGCCACTGATACAGGAGGCTACTCATGAACGAAAGACACTTAACctgtctcttata                                                                                                                                                                                                                                                                                                          >  1:463215/1‑289`  
             `CTGCAAACTGTGGATTACCAAGACCGAAGAGATGCTCGTAGAGAAAGGCTTGAAGCATGGCTGGGATGGGGACTTTGCGTACATGGCATGGGTACATGATGAAATCCAAGTAGGCTGCCGTACCGAAGAGATTGCTCAGGTGGTCATTGAGACCGCACAAGAAGCGATGCGCTGGGTTGGAGACCACTGGAACTTCCGGTGTCTTCTGGATACCGAAGGTAAGATGGGTCCTAATTGGGCGATTTGCCACTGATACAGGAGGCTACTCATGAACGAAAGACACTTAACAGGTGCGATTGTC                                                                                                                                                                                                                                                                                                         >  1:469604/1‑301`  
                 `AAACTGTGGATTATCAAGACCGAAGAGATGCTCGTAGAGAAAGGCTTGAAGCATGGCTGGGATGGGGACTTTGCGTACATGGCATGGGTACATGATGAAATCCAAGTAGGCTGCCGTACCGAAGAGAATGCTCAGGTGGTCATTGAGACCGCACAAGAAGCGATGCGCTGGGTTGGAGACCACTGGAACTTCCGGTGTCTTCTGGATACCGAAGGTAAGATGGGTCCTAATTGGGCGATTTGCTACTGATACAGGAGGCTACCCATGAACGAAAGACACTTAACAGGTGCGATCGTCTCCc                                                                                                                                                                                                                                                                                                     >  1:443609/1‑300`  
                    `CTGTGGATTATCAAGACCGAAGAGATGCTCGTAGAGAAAGGCTTGAAGCATGGCTGGGATGGGGACTTTGCGTACATGGCATGGGTACATGATGAAATCCAAGTAGGCTGCCGTACCGAAGAGATTGCTCAGGTGGTCATTGAGACCGCACAAGAAGCGATGCGCTGGGTTGGAGACCACTGGAACTTCCGGTGTCTTCTGGATACCGAAGGTAAGATGGGTCCTAATTGGGCGATTTGCCACTGATACAGGAGGCTACTCATGAACGAAAGACACTTAACAGGTGCGATTctgtctctta                                                                                                                                                                                                                                                                                                  >  1:610011/1‑291`  
                     `TGTGGATTATCAAGACCGAAGAGATGCTCGTAGAGAAAGGCTTGAAGCATGGCTGGGATGGGGACTTTGCGTACATGGCATGGGTACATGATGAAATCCAAGTAGGCTGCCGTACCGAAGAGATTGCTCAGGTGGTCATTGAGACCGCACAAGAAGCGATGCGCTGGGTTGGAGACCACTGGAACTTCCGGTGTCTTCTGGATACCGAAGGTAAGATGGGTCCTAATTGGGCGATTTGCCACTGATACAGGAGGCTACTCATGAACGAAAGACACTTAACAGGTGCGATTGTCCCCAATGG                                                                                                                                                                                                                                                                                                 <  1:117865/301‑1`  
                            `TATCAAGACCGAAGAGATGCTCGTAGAGAAAGGCTTGAAGCATGGCTGGGATGGGGACTTTGCGTACATGGCATGGGTACATGATGAAATCCAAGTAGGCTGCCGTACCGAAGAGATTGCTCAGGTGGTCATTGAGACCGCACAAGAAGCGATTCGCTGGGTTGGAGACCACTGGAACTTCCGGTGTCTTCTGGATACCGAAGGTAAGATGGGTCCTAATTGGGCGATTTGCCACTGATACAGGAGGCTACTCATGAACGAAAGACACTTAACAGGTGCGATTGTCCCCAATGGTAAACAG                                                                                                                                                                                                                                                                                          >  1:424658/1‑301`  
                            `TATCAAGACCGAAGAGATGCGCTGAGAGAAAGGCTTGAAGAATGGCTGGGATGGGGACTTTGCGTACATGGAATGGGTACATGATGAAATCCAAGTAGGCTGCCGTACCGAAGAGATTGCTCAGGTGGTCATTGAGACCGCACAAGAAGCGATGCGCTGGGTTGGAGACCACTGGAACTTCCGGTGTCTTCTGGATACCGAAGGTAAGATGGGTCCTAATTGGGCGATTTGCCACTGATACAGGAGGCTACTCATGAACGAAAGACACTTAACAGGTGCGATTGTCCCCAATGGTAAACAG                                                                                                                                                                                                                                                                                          <  1:51033/301‑1`  
                                 `AGACCGAAGAGATGCTCGTAGAGAAAGGCTTGAAGCATGGCTGGGATGGGGACTTTGCGTACATGGCATGGGTACATGATGAAATCCAAGTAGGCTTCCGTACAGAAGAGATTGCTCAGGTGGTCATTGAGACCGCACAAGAAGCGATGCGCTGGGTTGGAGACCACTGGAACTTCCGGTGTCTTCTGGATACCGAAGGTGAGATGGGTCCTAATTGGGTGATTTGCCACTGATACAGGAGGCTACTCATGAACTACAGACACTTACAAGGTGCGATTTTCCTCTATTGTAAACAGAAGGA                                                                                                                                                                                                                                                                                     >  1:67591/1‑301`  
                                 `AGACCGAAGAGATGCTCGTAGAGAAAGGCTTGAAGCATGGCTGGGATGGGGACTTTGCGTACATGGCATGGGTACATGATGAAATCCAAGTAGGCTGCCGTACCGAAGCGATTGCTCAGGTGGTCATTGAGACCGCACAAGAAGCGATGCGCCGGGTTGGAGACCCCTGGAACTTCCGGTGTCTTCTGGATACCGAAGGTAAGATGGGTCATAATTGGGCGATTTGCCACTGATACAGGAGGCTACTCATGAACGCACGCCACTTAACAGGTGCGATTGTCCCCAATGGTAAACAGAAGGA                                                                                                                                                                                                                                                                                     >  1:389507/1‑301`  
                                    `CCGAAGAGATGCTCGTAGAGAAAGGCTTGAAGCATGGCTGGGATGGGGACTTTGCGTACATGGCATGGGTACATGATGAAATCCAAGTAGGCTGCCGTACCGAAGAGATTGCTCAGGTGGTCATTGAGACCGCACAAGAAGCGATGCGCTGGGTTGGAGACCACTGGAACTTCCGGTGTCTTCTGGATACCGAAGGTAAGATGGGTCCTAATTGGGCGATTTGCCACTGATACAGGAGGCTACTCATGAACGAAAGACACTTAACAGGTGCGATTGTCCCCAATGGTAAACAGAAGGAAAT                                                                                                                                                                                                                                                                                  >  1:50069/1‑301`  
                                    `CCGAAGAGATGCTCGTAGAGAAAGGCTTGAAGCATGGCTGGGATGGGGACTTTGCGTACATGGCATGGGTACATGATGAAATCCAAGTAGGCTGCCGTACCGAAGAGATTGCTCAGGTGGTCATTGAGACCGCACAAGAAGCGATGCGCTGGGTTGGAGACCACTGGAACTTCCGGGGTCTTCTGGATACCGAAGGTAAGATGGGTCCTAATTGTGCGATTTGCCACTGATACAGGAGGCTACTCATGAACGAAAGACACTTAACAGGTGCGATTGTCCCCACTGGTAAACAGAAGTAAAT                                                                                                                                                                                                                                                                                  >  1:630316/1‑301`  
                                      `GAAGAGATGCTCGTAGAGAAAGGCTTGAAGCATGGCTGGGATGGGGACTTTGCGTACATGGCATGGGTACATGATGAAATCCAAGTAGGCTGCCGTACCGAAGAGATTGCTCAGGTGGTCATTGAGACCGCACAAGAAGCGATGCGCTGGGTTGGAGACCACTGGAACTTCCGGTGTCTTCTGGATACCGAAGGTAAGATGGGTCCTAATTGGGCGATTTGCCACTGATACAGGAGGCTACTCATGAACGACAGACACTTAACCGGTGCGATTGTCCCCAATGGTAAAAAG‑AGGAAAAGAa                                                                                                                                                                                                                                                                               >  1:162262/1‑300`  
                                      `GAAGAGATGCTCGTAGAGAAAGGCTTGAAGCATGGCTGGGATGGGGACTTTGCGTACATGGCATGGGTACATGATGAAATCCAAGTAGGCTGCCGTACCGAAGAGATTGCTCAGGTGGTCATTGAGACCGCACAAGAAGCGATGCGCTGGGTTGGAGACAACTGGAACTTCCGGTGTCTTCTGGATACCGAAGGTAAGATGGGTCCTAATTGGGCGATTTGCCACTGATACAGGAGGCTACTCATGAACGAAAGACACTTAACAGGTGCGATTGTCCCCAATGGTAAACctgtctcttata                                                                                                                                                                                                                                                                                >  1:123507/1‑289`  
                                       `tgtataagagacagGAGAAAGGCTTGAAGCATGGCTGGGATGGGGACTTTGCGTACATGGCATGGGTACATGATGAAATCCAAGTAGGCTGCCGTACCGAAGAGATTGCTCAGGTGGTCATTGAGACCGCACAAGAAGCGATGCGCTGGGTTGGAGACCACTGGAACTTCCGGTGTCTTCTGGATACCGAAGGTAAGATGGGTCCTAATTGGGCGATTTGCCACTGATACAGGAGGCTACTCATGAACGAAAGACACTTAACAGGTGCGATTGTCCCCAATGGTAAACAGAAGGAAATGAT                                                                                                                                                                                                                                                                               <  1:666977/287‑1`  
                                            `tttCTCGTAGAGAAAGGCTTTAAGCATGGCTGGGATGGGGACTTTGCGTACATGGCATGGGTACATTATGATATCCAAGTAGGCTGCCGTACCGAAGAGATTGCTCAGGTGGTCATTGAGACCGCACAAGAAGCGATGCGCTGGGTTGGAGACCACTGGAACTTCCGGTGTCTTCTGGATACCGAAGGTAAGATGGGTCCTAATTGGGCGATTTGCCACTGATACAGGAGGCTACTCATGAACGAAAGACACTTAACAGGTGCGATTGTCCCCAATGGTAAACAGAAGGAAATGATTGTCC                                                                                                                                                                                                                                                                          <  1:558455/298‑1`  
                                                     `GAGAAAGGCTTGAAGCATGGCTGGGATGGGGACTTTGCGTACATGGCATGGGTACATGATGAAATCCAAGTAGGCTGCCGTACCGAAGAGATTGCTCAGGTGGTCATTGAGACCGCACAAGAAGCGATGCGCTGGGTTGGAGACCACTGGAACTTCCGGTGTCTTCTGGATACCGAAGGTAAGATGGGTCCTAATTGGGCGATTTGCCACTGATACAGGAGGCTACTCATGAACGAAAGACACTTAACAGGTGCGATTGTCCCCAATGGTAAACAGAAGGAAATGATTGTCCAGTTCCTGA                                                                                                                                                                                                                                                                 >  1:178851/1‑301`  
                                                            `GCTTGAAGCATGGCTGGGATGGGGACTTTGCGTACATGGCATGGTTACATGATGAAATCCAAGTAGGCTGCCGTACCGAAGAGATTGCTCAGGTGGTCATTGAGACCGCACAAGAAGCGATGCGCTGGGTTGGAGACCACTGGAACTTCCGGTGTCTTCTGGAGACCGAAGGTAAGATGGGTCCTAATTGGGCGATTTGCCACTGATACAGGAGGCTACTCATGAACGAAAGACACTTAACAGGTGCGATTGTCCCCAATGGTAAACAGAAGGAAATGATTGTCCAGTTCCTGACACACG‑‑G                                                                                                                                                                                                                                                        <  1:297969/301‑1`  
                                                                  `AGCATGGCTGGGATGGGGACTTTGCGTACATGGCATGGGTACATGATGAAATCCAAGTAGGCTGCCGTACCGAAGAGATTGCTCAGGTGGTCATTGAGACCGCACAAGAAGCGATGCGCTGGGTTGGAGACCACTGGAACTTCCGGTGTCTTCTGGATACCGAAGGTAAGATGGGTCCTAATTGGGCGATTTGCCACTGATACAGGAGGCTACTCATGAACGAAAGACACTTAACAGGTGCGATTGTCCCCAATGGTAAACAGAAGGAAATGATTGTCCAGTTCCTGACACACG‑‑GTATGGA                                                                                                                                                                                                                                                  <  1:568790/301‑1`  
                                                                   `GCATGGCTGGGATGGGGACTTTGCGTACATGGCATGGGTACATGATGAAATCCAAGTAGGCTGCCGTACCGAAGAGATTGCTCAGGTGGTCATTGAGACCGCACAAGAAGCGATGCGCTGGGTTGGAGACCACTGGAACTTCCGGTGTCTTCTGGATACCGAAGGTAAGATGGGTCCTAATTGGGCGATTTGCCACTGATACAGGAGGCTACTCATGAACGAAGGACACTTAACAGGTGCGATTGTCCCCAATGGTAAACAGAAGGAAATGATTGTCCAGTTCCTGACACACG‑‑GTATGGAA                                                                                                                                                                                                                                                 <  1:366085/301‑1`  
                                                                    `CATGGCTGGGATGGGGACTTTGCGTACATGGCATGGGTACATGATGAAATCCAAGTAGGCTGCCGTACCGAAGAGATTGCTCAGGTGGTCATTGAGACCGCACAAGAAGCGATGCGCTGGGTTGGAGACCACTGGAACTTCCGGTGTCTTCTGGATACCGAAGGTAAGATGGGTCCTAATTGGGCGATTTGCCACTGATACAGGAGGCTACTCATGAACGAAAGACACTTAACAGGTGCGATTGTCCCCAATGGTAAACAGAAGGAAATGATTGTCCAGTTCCTGACACACG‑‑GTATGGAAG                                                                                                                                                                                                                                                <  1:544989/301‑1`  
                                                                    `CATGGCTGGGATGGGGACTTTGCGTACATGGCATGGGTACATGATGAAATCCAAGTAGGCTGCCGTACCGAAGAGAGTGCTCAGGTGGTCATTGAGACCGCACAAGAAGCGATGCGCTGGGTTGGAGACCACTGGAACTTCCGGTGTCTTCTGGATACCGAAGGTAAGATGGGTCCTAATTGGGCGATTTGCCACTGATACAGGAGGTTACTCATGAACGAAAGACACTTAACAGGTGCGATTGTCCCCAATGGTAAACAGAAGGAAATGATTGTCCAGTTCCTGACACACG‑‑GTATGGAAG                                                                                                                                                                                                                                                <  1:405677/301‑1`  
                                                                    `CATGGCTGGGATGGGGACTGTGAGTACAGGGCATGGGTACATGATGAAATCCAAGTAGGCTGCCGTACCGAAGAGATTTCTCAGGTGGTCATTGAGACCGCACAAGAAGCGATGCGCTGGGTTGGAGACCACTGGAACTTCCGGTGTCTTATGGATAACGAAGGTAAGATGGGTCCTAATTGGGCGATTTGCCAATGATACAGGAGGCTACTCATGAACGAAAGACACTTAACAGGTGCGATTGTCCCCAATGGTAAACAGAAGGAAATGATTGTCCAGTTCCTGACACACG‑‑GTATGGAAG                                                                                                                                                                                                                                                <  1:660976/301‑1`  
                                                                       `GGCTGGGATGGGGACTTTGCGTACATGGCATGGGTACATGATGAAATCCAAGTAGGCTGCCGTACCGAAGAGATTGCTCAGGTGGTCATTGAGACCGCACAAGAAGCGATGCGCTGGGTTGGAGACCACTGGAACTTCCGGTGTCTTCTGGATACCGAAGGTAAGATGGGTCCTAATTGGGCGATTTGCCACTGATACAGGAGGCTACTCATGAACGAAAGACACTTAACAGGTGCGATTGTCCCCAATGGTAAACAGAAGGAAATGATTGTCCAGTTCCTGCCACACG‑‑GTATGGAAGGAT                                                                                                                                                                                                                                             >  1:218385/1‑301`  
                                                                             `gacaGGGACTTTGCGTACATGGAATGGGTACATGATGAAATCCAAGTAGGCTGCCGTACCGAAGAGATTGACCAGGTGGTCATTGAGACCGAACAAGAAGCGATGCGGTGGGTTGGAGACCACTGGAACTTCCGGTGTCTTCTGGATACCGAAGGTAAGATGGGTCCTAATTGGGCGATTTGCCAATGATACAGGAGGCTAATAATGAACGAAAGACACTTAACAGGTGAGATTGTCCCCAATGGTAAACAGAAGGAAATGATTGTCCAGTTCCTGACACACG‑‑GTATGGAAGGATTGATGA                                                                                                                                                                                                                                       <  1:127655/297‑1`  
                                                                             `GATGGGGACTTTGCGTACATGGCATGGGTACATGATGAAATCCAAGTAGGCTGCCGTACCGAAGAGATTGCTCAGGTGGTCATTGAGACCGCACAAGAAGCGATGCGCTGGGTTGGAGACCACTGGAACTTCCGGTGTCTTCTGGATACCGAAGGTAAGATGGGTCCTAATTGGGCGATTTGCCACTGATACAGGAGGCTACTCATGAACGAAAGACACTTAACAGGTGCGATTGTCCCCAATGGTAAACAGAAGGAAATGATTGTCCAGTTCCTGACACACG‑‑GTATGGAAGGATTGATGA                                                                                                                                                                                                                                       <  1:564187/301‑1`  
                                                                                    `tataagagacagTGGCATGGGTACATGATGAAATCCAAGTATGCTGACGTAGCGAAGAGATTGCTCAGGTGGTCATTGAGACCGCACAAGAAGCGATGTGCTGGGTTGGAGACCACTGGAACTTCCGGTGTCTTCTGGATACCGAAGGTAAGATGGGTCCTAATTTGGCTATTTGCCACTGATACAGGAGGCTACTCATGAACGAAAGACACTTAACAGGTGCGATTGTCCCCAATGGTAAACAGAAGGAAATGATTGTCCAGTTCCTGACACACG‑‑GTATGGAAGGATTGATGACATTCGT                                                                                                                                                                                                                                <  1:634981/289‑1`  
                                                                                     `CTTTGCGTACATGGCATGGGTACATGATGAAATCCAAGTAGGCTGCCGTACCGAAGAGATTGCTCAGGTGGTCATTGAGACCGCACAAGCAACGATGCGCTGGGCTGGAGACCACTGGAACTTCCGGTGTCTTCTGGATACCGAAGGTAAGATGGGTCCTAATTGGGCGATTTGCCACTGATACAGGAGGCTACTCATGAACGAAAGACACTTAACAGGTGCGATTGTCCCCAATGGTAAACAGAAGGAAATGATTGTCCAGTTCCTGACACACG‑‑GTATGGAAGGATTGATGACATTCGTA                                                                                                                                                                                                                               >  1:395772/1‑301`  
                                                                                     `CTTTGCGTACATGGCATGGGTACATGATGAAATCCAAGTAGGCTGCCGTACCGAAGAGATTGCTCAGGTGGTCATTGAGACCACACAAGACGCGATGCGCTGGGTTGGAGACCACTGGAACTTCCGGTGTCTTCTGGATACCGAAGGTAAGATGGGTCCTAATTGGGCGATTTGCCACTGATACAGGAGGCTACTCATGAACGAAAGACACTTAACAGGTGCGATTGTCCCCAATGGTAAACAGAAGGAAATGATTGTCCAGTTCCTGACACACG‑‑GTATGGAAGGATTGATGACATTCGTA                                                                                                                                                                                                                               >  1:449385/1‑301`  
                                                                                      `TTTGCGTACATGGCATGGGTACATGATGAAATCCAAGTAGGCTGCCGTACCGAAGAGATTGCTCAGGTGGTCATTGAGACCGCACAAGAAGCGATGCGCTGGGTTGGAGACCACTGGAACTTCCGGTGTCTTCTGGATACCGAAGGTAAGATGGGTCCTAATTGGGCGATTTGCCACTGATACAGGAGGCTACTCATGAACGAAAGACACTTAACAGGTGCGATTGTCCCCAATGGTAAACAGAAGGAAATGATTGTCCAGTTCCTGACACACG‑‑GTATGGAAGGATTGATGACATTCGTAG                                                                                                                                                                                                                              <  1:111408/301‑1`  
                                                                                      `TTTGCGGACCTGGGATGGGGACATGATGAAAGCCAAGGAGGCTGACGTACCGAAGAGATTGCTCAGGTGGTAATTGAGACCGCACAAGAAGCGATGCGCTGGGTTGGAGACCACTGGAACTTCAGGGGTCTGCTGGATACCGAAGGTAAGATGGGTCCTAATTGGGCGATTGTCCAATGATACAGGAGGCTACTCATGAACGAAAGACACGTAACAGGTGCGATTGTCCCCAATGGTAAACAGAAGGAAAGGATTGTCCAGTTCCTGACACACG‑‑GTATGGAAGGATTGATGACATTCGTAG                                                                                                                                                                                                                              <  1:576675/301‑1`  
                                                                                         `GCGTACATGGCATGGGTACATGATGAAATCCAAGTAGGCTGCCGTACCGAAGAGATTGCTCAGGTGGTCATTGAGACCGCACAAGAAGCGATGCGCTGGGTTGGAGACCACTGGAACTTCCGGTGTCTTCTGGATACCGAAGGTAAGATGGGTCCTAATTGGGCGATTTGCCACTGATACAGGAGGCTACTCATGAACGAAAGACACTTAACAGGTGCGATTGTCCCCAATGGTAAACAGAAGGAAATGATTGTCCAGTTCCTGACACACG‑‑GTATGGAAGGATTGATGACATTCGTAGTAC                                                                                                                                                                                                                           >  1:343610/1‑301`  
                                                                                           `agatgtgaataagagACAGGATGAAATCCAAGTAGGCTGCCGTACCGAAGAGATTGCTCAGGTGGTCATTGAGACCGCACAAGAAGCGATGCGCTGGGTTGGAGACCACTGGAACTTCCGGTGTCTTCTGGATACCGAAGGTAAGATGGGTCCTAATTGGGCGATTTGCCACTGATACAGGAGGCTACTCATGAACGAAAGACACTTAACAGGTGCGATTGTCCCCAATGGTAAACAGAAGGAAATGATTGTCCAGTTCCTGACACACG‑‑GTATGGAAGGATTGATGACATTCGTAGTACGT                                                                                                                                                                                                                         <  1:455254/286‑1`  
                                                                                            `TACATGGCATGGGTACATGATGAAATCCAAGTAGGCTGCCGTACCGAAGAGATTGCTCAGGTGGTCATTGAGACCGCACAAGAAGCGATGCGCTGGGTTGGAGACCACTGGAACTTCCGGTGTCTTCTGGATACCGAAGGTAAGATGGGTCCTAATTGGGCGATTTGCCACTGATACAGGAGGCTACTCATGAACGAAAGACACTTAACAGGTGCGATTGTCCCCAATGGTAAACAGAAGGAAATGATTGTCCAGTTCCTGACACACG‑‑GTATGGAAGGATTGATGACATTCGTAGctgtct                                                                                                                                                                                                                        >  1:62339/1‑295`  
                                                                                              `gacaGCATGGGTACATGATGAAATCCAAGTAGGCTGCCGTACCGAAGAGATTGCTCAGGTGGTCATTGAGACCGCACAAGAAGCGATGCGCTGGGTTGGAGACCACTGGAACTTCCGGTGTCTTCTGGATACCGAAGGTAAGATGGGTCCTAATTGGGCGATTTGCCACTGATACAGGAGGCTACTCATGAACGAAAGACACTTAACAGGTGCGATTGTCCCCAATGGTAAACAGAAGGAAATGATTGTCCAGTTCCTGACACACG‑‑GTATGGAAGGATTGATGACATTCGTAGTACGTACA                                                                                                                                                                                                                      <  1:193326/297‑1`  
                                                                                                `TGGCATGGGTACATGATGAAATCCAAGTAGGCTGCCGTACCGAAGAGATTGCTCAGGTGGTCATTGAGACCGCACAAGAAGCGATGCGCTGGGTTGGAGACCACTGGAACTTCCGGTGTCTTCTGGATACCGAAGGTAAGATGGGTCCTAATTGGGCGATTTGCCACTGATACAGGAGGCTACTCATGAACGAAAGACACTTAACAGGTGCGATTGTCCCCAATGGTAAACAGAAGGAAATGATTGTCCAGTTCCTGACACACG‑‑GTATGGAAGGATTGATGACATTCGTAGTACGTACATC                                                                                                                                                                                                                    >  1:10579/1‑301`  
                                                                                                      `GGGTACATGATGAAATCCAAGTAGGCTGCCGTACCGAAGAGATTGCTCAGGTGGTCATTGAGACCGCACAAGAAGCGATGCGCTGGGTTGGAGACCACTGGAATTTCCGGTGTCTTCTGGATACCGAAGGTAAGATGGGTCCTAATTGGGCGATTTGCCACTGATACAGGAGGCTACTCATGAACGAAAGACACTTAACAGGTGCGATTGTCCCCAATGGTAAACAGAAGGAAATGATTGTCCAGTTCCTGACACACG‑‑GTATGGAAGGATTGATGACATTCGTAGTACGTACATCATctgt                                                                                                                                                                                                              >  1:172798/1‑297`  
                                                                                                      `GGGTACATGATGAAATCCAAGTAGGCTGCCGTACCGAAGAGATTGCTCAGGTGGTCATTGAGACCGCACAAGAAGCGATGCGCTGGGTTGGAGACCACTGGAACTTCCGGTGTCTTCTGGATACCGAAGGTAAGATGGGTCCTAATTGGGCGATTTGCCACTGATACAGGAGGCTACTCATGAACGAAAGACACTTAACAGGTGCGATTGTCCCCAATGGTAAACAGAAGGAAATGATTGTCCAGTTCCTGACACACG‑‑GTATGGAAGGATTGATGACATTCGTAGTACGTACATCATTTCG                                                                                                                                                                                                              <  1:549269/301‑1`  
                                                                                                               `ATGAAATCCAAGTAGGCTGCCGTACCGAAGAGATTGCTCAGGTGGTCATTGAGACCGCACAAGAAGCGATGCGCTGGGTTGGAGACCACTGGAACTTCCGGTGTCTTCTGGATACCGAAGGTAAGATGGGTCCTAATTGGGCGATTTGCCACTGATACAGGAGGCTACTCATGAACGAAAGACACTTAACAGGTTCGATTGTCCCCAATGGTAAACAGAAGGAAATGATTGTCCAGTTCCTGACACACG‑‑GTATGGAAGGATTGATGACATTCGTAGTACGTACATCATTTCGTGAGGCCAT                                                                                                                                                                                                     >  1:582831/1‑301`  
                                                                                                                    `ATCCAAGTAGGCTGCCGTACCGAAGAGATTGCTCAGGTGGTCATTGAGACCGCACAAGAAGCGATGCGCTGGGTTGGAGACCACTGGAACTTCCGGTGTCTTCTGGATACCGAAGGTAAGATGGGTCCTAATTGGGCGATTTGCCACTGATACAGGAGGCTACTCATGAACGAAAGACACTTAACAGGTGAGATTGTCCCCAATGGTAAACAGAAGGAAATGATTGTCCAGTTCCTGACACACG‑‑GTATGGAAGGATTGATGACATTCGTAGTACGTACATCATTTCGTGAGGCCATTAAGG                                                                                                                                                                                                >  1:533789/1‑301`  
                                                                                                                     `gtgtatacgagacagGTACCGAAGGGATTGCTCAGGTGGTCATTGAGACCGAAGAAGAAGCGATGCGCTGGGTTGGAGACCACTGGAACTTACGGTGTCTTCTGGATACCGAAGGTAAGATGGGTCCTAATTGGGCGATTTGCCACTGATACAGGAGGCTACTCATGAACGAAAGACACTTAACAGGTGCGATTGTCCCCAATGGTAAACAGAAGGAAATGATTGTCCAGTTCCTGACACACG‑‑GTATGGAAGGATTGATGACATTCGTAGTACGTACATCATTTCGTGAGGCCATTAAGGA                                                                                                                                                                                               <  1:402059/286‑1`  
                                                                                                                     `aCCAAGTAGGCTGCCGTACCGAAGGGATTGCTCAGGTGGTCATTGAGATCGGACAAGAAGCGATGCGCTGGGTTGGAGACCACTGGAACTTCCGGTGTATTCTGGATACCGAAGGTAAGATGGGTCCTAATTGGGCGATTTGCCACTGATACAGGAGGCTACTCATGAACGAGAGACACGTAACAGGTGCGATTGTCCCCAATGGTAAACAGAAGGAAATGATTGTCCAGTTCCTGACACACG‑‑GTATGGAAGGATTGATGACATTCGTAGTACGTACATCATTGCGTGAGGCCATTAAGGA                                                                                                                                                                                               <  1:356116/300‑1`  
                                                                                                                     `TCCAAGTAGGCTGTCGTACCGAAGAGATTGCTCAGGTGGTCATTGAGACCGCACAAGAAGCGATGCGCTGGGTTGGAGACCACTGGAACTTCCGGTGTCTTCTGGATACCGAAGGTAAGATGGGTCCTAATTGGGCGATTTGCCACTGATACAGGAGGCTACTCATGAACGAAAGACACTTAACAGGTGCGATTGTCCCCAATGGTAAACAGAAGGAAATGATTGTCCAGTTCCTGACACACG‑‑GTATGGAAGGATTGATGACATTCGTAGTACGTACActgtctcttatacacatctccga                                                                                                                                                                                               >  1:243482/1‑278`  
                                                                                                                                 `GCCGTACCGAAGAGATTTCTCAGGTGGTCATTGAGACCGCACAAGAAGCGATGCGCTGGGTTGGAGACCACTGGAACTTCCGGTGTCTTCTGGATACCGAAGGTAAGATGGGTCCTAATTGGGCGATTTGCCACTGATACAGGAGGCTACTCATGAACGAAAGACACTTAACAGGTGCGATTGTCCCCAATGGTAAACAGAAGGAAATGATTGTCCAGTTCCTGACACACG‑‑GTATGTAAGGATTGATGACATTCGTAGTACGTACATCATTTCGTGAGGCCTTTAAGGACATGCACGAAGA                                                                                                                                                                                   >  1:198176/1‑301`  
                                                                                                                                       `CCGAAGAGATTGCGCAGGTGGTCATTGAGACCGCACAAGAAGCGATGCGCTGGGTTGGAGACCACTGGAACTTCCGGTGTCTTCTGGATACCGAAGGTAAGATGGGTCCTAATTGGGCGATTTGCCACTGATACAGGAGGCTACTCATGAACGAAAGACACTGAACAGGTGCGATTGTCCCCAATGGTAAACAGAAGGAAATGATTGTCCAGTTCCTGACACACG‑‑GTATGGAAGGATTGATGACATTCGTAGTACGTACATCATTTCGTGAGGCCATTAAGGACATGCACGAAGAGTATGC                                                                                                                                                                             <  1:642486/301‑1`  
                                                                                                                                       `CCGAAGAGATTGATCAGGTGGTAAGTGAGACCGAACAAGAAGCGATGCGCTGGGTTGGAGACCACTGGAACGTCCGGGGTCTTCTGGTTAACGAAGGTAAGATGGGTCATCATTGGGAGATTGGCCACTGATACAGGAGTCTGCTCATTAACGAAAAACACTTACCAGGTGCGATGTTCCCCAATGGGAAACAGAAGGAAATGATTGTCCGGTTCCCGACACACG‑‑GTATGGAAGGATTTAGGACATTCGTAGTACGTACATCATTTCGTGAGGCCAGTAAGGCCATGCACGAAGAGTATGC                                                                                                                                                                             <  1:648060/301‑1`  
                                                                                                                                          `gtatatgagacagGGTGGGCATTGAGACCGCACAAGAATCGATGCGCTGGGTTGGAGACCACTGGAACTTCCGGTGTTTTCTGGATACCGAAGGTATGATTGGTCCTAATTGGGCGATTTGCCACTGATACAGGAGGCTACTCATGAACGAAAGACACTTAACAGGTTCGATTGTCCCAAATGGTAAACAGAAGGAAATGATTGTCCAGTTCCTGACACACG‑‑GTATGGAAGGATTGATGACATTCGTAGTACGTACATCATTTCGTGAGGCCATTAAGGACATGCACGAAGAGTATGCAGA                                                                                                                                                                          <  1:318750/288‑1`  
                                                                                                                                          `AAGAGATTGCTCAGGTGGTCAGTGAGACCGCACAAGAAGCGATGCGCTGGGTTGGAGACCACTGGAACTTCCGGTGTCATTTGGATACAGAAGGTAAGATGGGTCCTAATTGGGCGATTTGCCACGGATACAGGAGGCTACTCAGGAACGAAAGACACTTAACAGGTGCGATTGTCCCCAATGGTAAACAGAAGGAAATGATTGTCCAGTTCCTGACACACG‑‑GTATGGAAGGATTGATGAAATTCGTAGTACGTACATCATTTCGTGAGGCCATTAAGGACATGCACGAAGAGTATGCAGA                                                                                                                                                                          <  1:484400/301‑1`  
                                                                                                                                          `AAGAGAGTGCTCAGGTGGTCATTGAGACCGCACAAGAAGCGATGCGCTGGGTTGGAGACCACTGGAACTTCCGGTGTATTCTGGATAACGAAGGTAAGATGGGTCCTAATTGGGCGATTTGCCACTGATACAGGAGGCTACTCATGAACGAAAGACACTTAACAGGTGCGATTGTCCCCAATGGTAAACAGAAGGAAATGATTGTCCAGTTCCTGACACACG‑‑GTATGGAAGGATTGATGACATTCGTAGTACGTACATCATTTCGTGAGGCCATTAAGGACATGCACGAAGAGTATGCAGA                                                                                                                                                                          <  1:553372/301‑1`  
                                                                                                                                                  `tCTCAGGTGGTCATTGAGACCGCACAAGAAGCGATGCGCTGGGTTGGAGACCACTGGAACTTCCGGTGTCTTCTGGATACCGAAGGTAAGATGGGTCCTAATTGGGCGATTTGCCACTGATACAGGAGGCTACTCATGAACGAAATACACTTAACAGGTGCGATTGTCCCCAATGGTAAACAGAAGGAAATGATTGTCCAGTTCCTGACACACG‑‑GTATGGAAGGATTGATGACATTCGTAGTACGTACATCATTTCGTGAGGCCATTAAGGACATGCACGAAGAGTATGCAGATAAGGACT                                                                                                                                                                  <  1:145419/300‑1`  
                                                                                                                                                        `GTGGTCATTGAGACCGCACAAGAAGCGATGCGCTGGGTTGGAGACCACTGGAACTTCCGGTGTCTTCTGGATACCGAAGGTAAGATGGGTCCTAATTGGGCGATTTGCCACTGATACAGGAGGCTACTCATGAACGAAAGACACTTAACAGGTGCGATTGTCCCCAATGGTAAACAGAAGGAAATGATTGTCCAGTTCCTGACACATG‑‑GTATGGAAGGATTGATGACATTCGTAGTACGTACATCATTTCGTGAGGCCATTAAGGACATGCACGAAGAGTATGCAGATAAGGACctgtctt                                                                                                                                                            >  1:324383/1‑294`  
                                                                                                                                                          `GGTCATTGAGACCGCACAAGAAGCGATGCGCTGGGTTGGAGACCACTGGAACTTCCGGTGTCTTCTGGATACCGAAGGTAAGATGGGTCCTAATTGGGCGATTTGCCACTGATACAGGAGGCTACTCATGAACGAAAGACACTTAACAGGTGCGATTGTCCCCAATGGTAAACAGAAGGAAATGATTGTCCAGTTCCTGACACACG‑‑GTATGGAAGGATTGATGACATTCGTAGTACGTACATCATTTCGTGAGGCCATTAAGGACATGCACGAAGAGTATGCAGctgtctcttatacacat                                                                                                                                                          >  1:337179/1‑284`  
                                                                                                                                                          `GGTCATTGAGACCGCACAAGAAGCGATGCGCTGGGTTGGAGACCACTGGAACTTCCGGTGTCTTCTGGATACCGAAGGTAAGATGGGTCCTAATTGGGCGATTTGCCACTGATACAGGAGGCTACTCATGAACGAAAGACACTTAACAGGTGCGATTGTCCCCAATGGTAAACAGAAGGAAATGATTGTCCAGTTCCTGACACACG‑‑GTATGGAAGGATTGATGACATTCGTAGTACGTACATCATTTCGTGAGGCCATTAAGGACATGCACGAAGAGTATGCAGATAAGGACTCTTTCAAA                                                                                                                                                          >  1:350439/1‑301`  
                                                                                                                                                           `GTCATTGAGACCGCACAAGAAGCGATGCGCTGGGTTGGAGACCACTGGAACTTCCGGTGTCTTCTGGATACCGAAGGTAAGATGGGTCCTAATTGGGCGATTTGCCACTGATACAGGAGGCTACTCATGAACGAAAGCCACTTAACAGGTGCGATTGTCCCCAATGGTAAACAGAAGGAAATGATTGTCCAGTTCCTGACACACG‑‑GTATGGAAGGATTGATGACATTCGTAGTACGTACATCATTTCGTGAGGCCATTAAGGACATGCACGAAGAGTATGCAGATAAGGACTCTTTCAAAC                                                                                                                                                         >  1:291180/1‑301`  
                                                                                                                                                           `GTCATTGAGACCGAACAAGAAGCGATGCGCTGGGTTGGAGACCACTGGAACTTCCGGTGTCTTCTGGATACCGAAGGTAAGATGGGTCCTAATTGGGCGATTTGCCACTGATACAGGAGGCTACTCATGAACGAAAGACACTTAACAGGTGCGATTGTCCCCAATGGTAAACAGAAGGAAATGATTGTCCAGTTCCTGACACACG‑‑GTATGGAAGGATTGATGACATTCGTAGTACGTACATCATTTCGTGAGGCCATTAAGGACATGCACGAAGAGTATGCAGATAAGGACTCTTTCAAAC                                                                                                                                                         <  1:43748/301‑1`  
                                                                                                                                                           `GTCATTGAGACCGAACAAAACGCGATGCGGTGGGTTGGAGACCACTGGAACTTCCGGTGTCTTCTGGATACCGAAGGTAAGATGGGTCCTAATTGGGCGATTTGCCACTGATACAGGAGGCTACTCATGAACGAAAGACACTTACCAGGTGCGATTGTCCCCAATGGTAAACAGAAGGAAATGATTGTCCAGTTCCTGACACACG‑‑GTATGGAAGGATTGATGACATTCGTAGTACGTACATCATTTCGTGAGGCCATTAAGGACATGCACGAAGAGTATGCAGATAAGGACTCTTTCAAAC                                                                                                                                                         <  1:47977/301‑1`  
                                                                                                                                                                     `CCGGACAAGAAGCGATGCGCTGGGTTGGAGACCACTGGAACTTCCGGTGTCTTCTGGATGCCGAAGGTAAGATGGGTTCTAATTGGGCGATTTGCCACTGTTACAGGAGGCTACTCATGAACGAAAGACACTTAACAGGTGCGATTGTCCCCAATGGTAAACAGAAGGAAATGATTGTCCAGTTCCTGACACACG‑‑GTATGGAGGGATTGATGACATTCGTAGTACGTACATCATTTCGTGAGGCCATTAAGGACATGCACGAAGAGTATGCAGATAAGGACTCTTTCAAACAATCTCCTGC                                                                                                                                               <  1:474419/301‑1`  
                                                                                                                                                                        `CACTAGAAGCGATGCGCTGGGTTGGAGACCACTGGAACTTCCGGTGTCTTCTGGATACCGAAGGTAAGATGGGTCCTAATTGGTCGATTTGCCACTGATACAGGAGGCTACTCATGAACGAAAGACACTTAACAGGTGCGATTGTCCCCAATGGTAAACAGAAGGAAATGATTGTCCAGTTCCTGACATACG‑‑GTATGGAAGGATTGATGACATTCGTAGTACGTACATCATTTCGTGAGTCCATTAAGGACATGCACGAAGAGTATGCAGATAAGGACTCTTTCAAACAATCTCCTGCAAC                                                                                                                                            <  1:166254/301‑1`  
                                                                                                                                                                            `AGAAGCGATGCGCTGGGTTGGAGACCACTGGAACTTCCGGTGTCTTCTGGATACCGAAGGTAAGATGGGTCCTAATTGGGCGATTTGCCACTGATACAGGAGGCTACTCATGAACGAAAGACACTTAACAGGTGCGATTGTCCCCAATGGTAAACAGAAGGAAATGATTGTCCAGTTCCTGACACACG‑‑GTATGGAAGGATTGATGACATTCGTAGTACGTACATCATTTCGTGAGGCCATTAAGGACATGCACGAAGAGTATGCAGATAAGGACTCTTTCAAACAATCTCCTGCAACAGTA                                                                                                                                        >  1:152540/1‑301`  
                                                                                                                                                                                `GCGATGCGCTGGGTTGGAGACCACTGGAACTTCCGGTGTCTTCTGGATACCGAAGGTAAGATGGGTCCTAATTGGGCGATTTGCCACTGATACAGGAGGCTACTCATGAACGAAAGACACTTAACAGGTGCGATTGTCCCCAATGGTAAACAGAAGGAAATGATTGTCCAGTTCCTGACACACG‑‑GTATGGAAGGATTGATGACATTCGTAGTACGTACATCATTTCGTGAGGCCATTAAGGACATGCACGATGAGTATTCAGATAAGGACTCTTTCctgtcccttatacacatctccgaac                                                                                                                                    >  1:49462/1‑276`  
                                                                                                                                                                                   `ATGCGCTGGGTTGGAGACCACTGGAACTGCCGGTGACGTCTGGATACCGAAGGTAAGATGGGTCCTAATTGGGCGATTTGCCACTGATACAGGAGGCTACTCATGAACGAAAGACACTTAACAGGTGCGATTGTCCCCAATGGTAAACAGAAGGAAATGATTGTCCAGTTCCTGACACACG‑‑GTATGGAAGGATTGATGACATTCGTAGTACGTACATCATTTCGTGAGGCCATTAAGGACATGCACGAAGAGTATGCAGATAAGGACTCTTTCAAACAATCTCCTGCAACAGTACGGGAGG                                                                                                                                 <  1:30475/301‑1`  
                                                                                                                                                                                    `TGCGCTGGGTTGGAGACCACTGGAACTTCCGGTGTCTTCTGGATACCGAAGGTAAGATGGGTCCTAATTGGGCGATTTGCCACTGATACAGGAGGCTACTCATGAACGAAAGACACTTAACAGGTGCGATTGTCCCCAATGGTAAACAGAAGGAAATGATTGTCCAGTTCCTGACACACG‑‑GTATGGAAGGATTGATGACATTCGTAGTACGTACATCATTTCGTGAGGCCATTAAGGACATGCACGAAGAGTATGCCGATAAGGACTCTTTCAAACAATATCCTGCAACAGTACGGGAGGT                                                                                                                                >  1:100060/1‑301`  
                                                                                                                                                                                           `GGTTGGAGACCACTGGAACTTCCGGTGTCTTCTGGATACCGAAGGTAAGATGGGTCCTAATTGGGCGATTTGCCACTGATACAGGAGGCTACTCATGAACGAAAGACACTTAACAGGTGCGATTGTCCCCAATGGTAAACAGAAGGAAATGATTGTCCAGTTCCTGACACACG‑‑GTATGGAAGGATTGATGACATTCGTAGTACGTACATCATTTCGTGAGGCCATTAAGGACATGTACGAAGAGTATGCAGATAAGGACTCTTTCAAACAATCTCCTGCAACAGTACGGGCGGTctgtctc                                                                                                                         >  1:268760/1‑294`  
                                                                                                                                                                                               `GGAGACCACTGGAACTTCCGGTGTCTTCTGGATACCGAAGGTAAGATGGGTCCTAATTGGGCGATTTGCCACTGATACAGGAGGCTACTCATGAACGAAAGACACTTAACAGGTGCGATTGTCCCCAATGGTAAACAGAAGGAAATGATTGTCCAGTTCCTGACACACG‑‑GTATGGAAGGATTGATGACATTCGTAGTACGTACATCTTTTCGTGAGGCCATTAAGGACATGCACGAAGAGTATGCAGATAAGGACTCTTTCAAACAATCTCCTGCAACAGTACGGGAGGTGTTCTGATGct                                                                                                                     >  1:522071/1‑299`  
                                                                                                                                                                                                    `CCACTGGAACTTCCGGTGTCTTCTGGATACCGAAGGAAAGATGGGTCCTAATTGGGCGATTTGCCACTGATACAGGAGGCTACTCATGAACGAAAGACACTTAACAGGTGCGATTGTCCCCAATGGTAAACAGAAGGAAATGATTGTCCAGTTCCTGACACACG‑‑GTATGGAAGGATTGATGACATTCGTAGTACGTACATCATTTCGTGAGGCCATTAAGGACATGCACGAAGAGTATGCAGCTAAGGACTCTTTCAAACAATCTCCTGCAACAGTACGGGAGGTGTTCTGATGTCTGACT                                                                                                                >  1:261804/1‑301`  
                                                                                                                                                                                                     `CACTGGAACTTCCGGTGTCTTCTGGATACCGAAGGTAAGATGGGTCCTAATTGGGCGATTTGCCACTGATACAGGAGGCTACTCATGAACGAAAGACACTTAACAGGTGCGATTGTCCCCAATGGTAAACAGAAGGAAATGATTGTCCAGTTCCTGACACACG‑‑GTATGGAAGGATTGATGACATTCGTAGTACGTACATCATTTCGTGAGGCCATTAAGGACATGCACGAAGAGTATGCAGGTAAGGACTCTTTCAAACAATCACATGCAACAGTACGGGAGGTGTTCTGATGctgtctct                                                                                                               >  1:516441/1‑293`  
                                                                                                                                                                                                                     `GTCTTCTGGATACCGAAGGTAAGATGGGTCCTAATTGGGCGATTTGCCACTGATACAGGAGGCTACTCATGAACGAAAGACACTTAACAGGTGCGATTGTCCCCAATGGTAAACAGAAGGAAATGATTGTCCAGTTCCTGACACACG‑‑GTATGGAAGGATTGATGACATTCGTAGTACGTACATCATTTCGTGAGGCCATTAAGGACATGCACGAAGAGTATGCAGATAAGGACTCTTTCAAACAATCTCCTGCAACAGTACGGGAGGTGTTCTGATGTCTGACTACCTGACAGTGCTGCAA                                                                                               >  1:256935/1‑301`  
                                                                                                                                                                                                                                 `CCGAAGGTAAGATGGGTCCTAATTGGGCGATTTGCCACTGATACAGGAGGCTACTCATGAACGAAAGACACTTAACAGGTGCGATTGTCCCCAATGGTAAACAGAAGGAAATGATTGTCCAGTTCCTGACACACG‑‑GTATGGAAGGATTGATGACATTCGTAGTACGTACATCATTTCGTGAGGCCATTAAGGACATGCACGAAGAGTATGCAGATAAGGACTCTTTCAAACAATCTCCTGCAACAGTACGGGAGGTGTTCTGATGTCTGACTACCTGAAAGTGCTGCAAGCAATCAAAAGT                                                                                   <  1:41118/301‑1`  
                                                                                                                                                                                                                                      `GGTAAGATGGGTCCTAATTTGGCGATTTGCCACTGATACAGGAGGCTACTCATGAACGAAAGACACTTAACAGGTGCGATTGTCCCCAATGGTAACCAGAAGGAAATGATTGTCCAGTTCCTGACACACG‑‑GTATGGAAGGCTTGATGACATTCGTAGTACGTACATCATTTCGTGAGGCCATTAAGGACATGCACGAAGAGTATGCAGATAAGGACTCTTTCAAACAATCACCTGCAACAGTACGGGCGGTGTTCTGATGTCTGACTACCTGAAAGTGCTGCAAGCAATCAAAAGTCGCCC                                                                              >  1:378782/1‑301`  
                                                                                                                                                                                                                                      `GGTAAGATGGGTCCTAATTGGGCGATTTGCCACTGATACAGGAGGCTACTCATGAACGAAAGACACTTAACAGGTTCGATTGTCCCCAATGGTAAACAGAAGTAAATGATTGTCCATTTCCTGACACCCT‑‑GTATGGAAGGATTTATGACATTCGTAGTACGTACATCATTTCTTTAGGCCATTAAGGACATGCACGAAGAGTATGCAGATAAGTACTCTTTCATACAATCTCCTGCAACAGTACGGGAGGTGTTCTGATGTCTTACTACCTGAAATTTCTGCAAGCAATCAAAATTTGCCt                                                                              >  1:331432/1‑300`  
                                                                                                                                                                                                                                            `agGGGTCCTAATTGGGCGATTTGCCACTGATACAGGAGGCTACTCATGAACGAAAGACACTTAACAGGTGCGATTGTCCCCAATGGTAAAAAGAAGGAAATGATTGTCCAGTTCCTGACACACG‑‑GTATGGAAGGATTGATGACATTCGTAGTACGTACATCATTTCGTGAGGCCATTAAGGACATGCACGAAGAGTATGCAGATAAGGACTCTTTCAAACAATCTCCTGCAACAGTACGGGAGGTGTTCTGATGTCTGACTACCTGAAAGTGCTGCAAGCAATCAAAAGTTGCCCTAAGAC                                                                        <  1:272599/299‑1`  
                                                                                                                                                                                                                                            `aaGGGTACTAAGTGGGCGATTGGACAAGGATAAAGGAAGCTAAGCATGAACGAAAGACACTTAACAGGTGCGATTGTCCCCAATGGTAAACAGAAGGAAATGATTGTCCAGTTCCTGAAACACG‑‑GTATGGAAGGATTGATGACATTCGTAGTACGTACATCATTTCGTGAGGCCATTAAGGACATGCACGAAGAGTATGCAGATAAGGACTCTTTCAAACAATCTCCTGCAACAGTACGGGAGGTGTTCTGATGTCTGACGACCTGAAAGTGCTGCAAGCAATCAAAAGTTGCCCTAAGAC                                                                        <  1:15742/299‑1`  
                                                                                                                                                                                                                                            `ATGGGTCCTAATTGGGCGATTTGCCACTGATACAGGAGGCTACTCATGAACGAAAGACACTTAACAGGTGCGATTGTCCCCAATGGTAAACAGAAGGAAATGATTGTCCAGTTCCTGACACACG‑‑GTATGGAAGGATTGATGACATTCGTAGTACGTACATCATTTCGTGAGGCCATTAAGGACATGCACGAAGAGTATGCAGATAAGGACTCTTTCAAACAATCTCCTGCAACAGTACGGGAGGTGTTCTGATGTCTGACTACCTGAAAGTGCTGCAAGCAATCAAAAGTTGCCCTAAGAC                                                                        <  1:250591/301‑1`  
                                                                                                                                                                                                                                              `GGGTCCTAATTGGGCGATTGGCCACGTATACAGTAGGCTACTCATGAAAGAAAGACACTTAACAGGTGCGATTGTCCCCAATGGTAAACAGAAGGAAATGATTGTCCAGTTCCTGACACACGGTGTATGGAAGGATTGATGACAGTCGTAGTACGTACATCATTTCGTGAGTCCATTAAGGACATGCACGAAGAGTATGCAGATAAGGACTCTTTCAAACAATCTCCTGCAACAGTACGGGATGTGTTCTGATGTCTGACTACCTGAAAGTGCTGCAAGCAATCAAAAGTTGCCCTAAGAC                                                                        <  1:655357/301‑1`  
                                                                                                                                                                                                                                                 `TCCTAATTGGGCGATTTGCCACTGATACAGGAGGCTACTCATGAACGAAAGACACTTAACAGGTGCGATTGTCCCCAATGGTAAACAGCAGGAAATGATTGTCCAGTTCCTGACACACT‑‑GTATGGAAGGATTGATGACATTCGTAGTACGTACATCATTTCGTGAGGCCATTAAGGACATGCACGAAGAGTATGCAGATAAGGACTCTTTCAAACAATCTCCTGCCACAGTACGGGAGGTGTTCTGATGTCTGACTACCTGACAGTGCTGCAAGCAATCAACAGTTGCCCctgtctcttat                                                                   >  1:92598/1‑290`  
                                                                                                                                                                                                                                                 `TCCTAATTGGGCGATTTGCCAATGATACAGGAGGCTAATCATGAACGAAAGACACTTAACAGGTGCGATTGTCCCCAATGGTAAACAGAAGGAAATGATTGTCCAGTTCCTGACACACG‑‑GTATGGAAGGATTGATGACATTCGTAGTACGTACATCATTTCGTGAGGCCATTAAGGACATGCACGAAGAGTATGCAGATAAGGACTCTTTCAAACAATCTCCTGCAACAGTACGGGAGGTGTTCTGATGTCTGACTACCTGAAAGTGCTGCAAGCAATCAAAAGTTGCCCTAAGACTTTCC                                                                   <  1:205420/301‑1`  
                                                                                                                                                                                                                                                    `TAATTGGGCGATTTGCCACTGATACAGGAGGCTACTCATGAACGAAAGACACTTAACAGGTGCGATTGTCCCCAATGGTAAACAGAAGGAAATGATTGTCCAGTTCCTGACACACG‑‑GTATGGAAGGATTGATGACATTCGTAGTACGTACATCATTTCGTGCGGCCATTAAGGACATTCACGAAGAGTATGCAGATAAGGACTCTTTCAAACAATCCCCTGCAACAGTACGGGAGGTGTTCTGATGTCTGACTACCTGAAAGTGCTGCTAGCAATCAAAAGTTTCCCTAAGCCTTTCCAGT                                                                >  1:361520/1‑301`  
                                                                                                                                                                                                                                                          `GGCGATTTGCCACTGATACAGGAGGCTACTCATGAACGAAAGACACTTAACAGGTGCGATTGTCCCCAATGGTAAACAGAAGGAAATGATTGTCCAGTTCCTGACACACG‑‑GTATGGAAGGATTGATGATATTCGTAGTACGTACATCTTTTCGTGTGGTCATTAAGGACATGCACGAAGAGTATGCAGACAAGGATTCTTTCAAACAATCCCCTGCAACAGTTCGGGAGGTGTTCTGATGTCTGACTACCTGAAATCGCTGCAAGCAATCAACAGTTGCCTTAAGCCTTTCCCTTCCTACT                                                          >  1:384306/1‑301`  
                                                                                                                                                                                                                                                                `agacagcaGATACAGGAGGCTACTCATGAACGAAAGACACTTAACAGGTGCGATTGTACACAAGGGTAAAGAGAAGGAAATGATTGTCCAGTTCATGACACACG‑‑GTATGGAAGGATTGATGACATTCGTAGTACGTACATCATTTCGTGAGGCAATTAAGGACATGCAAGAAGAGTATGCAGATAAGGACTCTTTAAAACAATCTCCTGCAACAGTACGGGAGGTGTTCTGATGTCTTACTACCTGAAAGTGCTGCAAGCAATCAAAAGTTGCCCTAAGACTTTCCAGTCCAACTATGTAC                                                    <  1:224400/293‑1`  
                                                                                                                                                                                                                                                                     `ggagatgtgtataagagacagTGAACGAAAGACACTTAACAGGTGCGATTGTCCCCAATGGTAAACAGAAGGAAATGATTGTCCAGTTCCTGACACACG‑‑GTATGGAAGGATTGATGACATTCGTAGTACGTACATCATTTCGTGAGGCCATTAAGGACATGCACGAAGAGTATGCAGATAAGGACTCTTTCAAACAATCTCCTGCAACAGTACTGGAGGTGTTCTGATGTCTGACTACCTGAAAGTGCTGCAAGCAATCAAAAGTTGCCCTAAGACTTTCCAGTCCAACTATGTACGGAAC                                               <  1:237121/280‑1`  
                                                                                                                                                                                                                                                                     `gctcggagatgtgtataagagacagCGAAAGACACTTAACAGGTGCGATTGTCCCCAATGGTAAACAGAAGGAAATGATTGTCCAGTTCCTGACACACG‑‑GTATGGAAGGATTGATGACATTCGTAGTACGTACATCATTTCGTGAGGCCATTAAGGACATGCACGAAGAGTATGCAGATAAGGACTCTTTCAAACAATCTCCTGCAACAGTACGGGAGGTGTTCTGATGTCTGACTACCTGAAAGTGCTGCAAGCAATCAAAAGTTGCCCTAAGACTTTCCAGTCCAACTATGTACGGAAC                                               <  1:132956/276‑1`  
                                                                                                                                                                                                                                                                     `aagGATAAAGGAGGCGACTCATGAAAGAAAGACACTGAACAGAGTCGATTGTACCCAATAGTAAACAAAAGGAAATGATAGTCCAGTTCCTGACACACG‑‑GTATGGAAAGATTGATTACATTCGTAGTACGTACATCATTTCGTGAGGCCATTAAGGACATGCACGAAGAGTATGCAGATAAGGACTCTTTCAAACAATCTCCTGCAACAGTACGGGAGGTGTTCTGATGTCTGACTACCTGAAAGTGCTGCAAGCAATCAAAAGTTGCCCTAAGACTTTCCAGTCCAACTATGTACGGAAC                                               <  1:590605/298‑1`  
                                                                                                                                                                                                                                                                      `aTGATACAGGAGGCTACTCATGAACGAAAGACACTTAACAGGTGCGATTGTCCCCAATGGTAAACAGAAGGAAATGATTGTCCAGTTCCTGACACACG‑‑GTATGGAAGGATTGATGACATTCGTAGTACGTACATCATTTCGTGAGGCCATTAAGGACATGCACGAAGAGTATGCAGATAAGGACTCTTTCAAACAATCTCCTGCAACAGTCCGGGAGGTGTTCTGATGTCTGACTACCTGAAAGAGCTGCAAGCAATAAAAAGTTGCCCTATGACTTTACAGTCCAACTATGTACGGAACA                                              >  1:598614/2‑301`  
                                                                                                                                                                                                                                                                      `CTGATACAGGAGGCTACTCCTGAACGAAAGACACTTAACAGGTGCGATTGTCCCCAATGGTAAACTGAAGGAAATGATTGTCCAGTTCCTGACACTCG‑‑GTATGGAAGGATTGATGACATTCGTAGTACGTACATCATTTCGTGAGGCCATTTAGGACATGCACGAAGCGTATGCAGATAAGGACTCTTTCAAACAAACTCCTGCAACAGTACGGGAGGTGTTCTGATGTCTGACTACCTGAAAGTGCTGCAAGCAATTAAAAGTTGCCCTAAGACTTTCCAGCCCAACTATGTACGGAACA                                              >  1:605475/1‑301`  
                                                                                                                                                                                                                                                                         `ATACAGGAGGCTACTCATGAACGAAAGACACTTAACAGGTGCGATTGTCCCCAATGGTAAACAGAAGGAAATGATTGTCCAGTTCCTGACACACG‑‑GTATGGAAGGATTGATGACATTCGTAGTACGTACATCATTTCGTGAGGCCATTAAGGACATGCACGAAGAGTATGCAGATAAGGACTCTTTCAAACAATCTCCTGCAACAGTACGGGAGGTGTTCTGATGTCTGACTACCTGAAAGTGCTGCAAGCAATCAAAAGTTGCCCTAAGACTTTCCAGTCAAACTATGTACGGAACCATG                                           >  1:311710/1‑301`  
                                                                                                                                                                                                                                                                                 `GGCTACTCATGAACGAAAGACACTTAACAGGTGCGATTGTCCCCAATGGTAAACAGAAGGAAATGATTGTCCAGTTCCTGACACACG‑‑ATATGGAAGGATTGATGACATTCGTAGTACGTACATCATTTCGTGAGGCCATTAAGGACATGCACGAAGAGTATGCAGATAAGGACTCTTTCAAACAATCTCCTGCAACAGTACGGGAGGTGTTCTGATGTCTGACTACCTGAAAGTGCTGCAAGCAATCAAAAGTTGCCCTAAGACTTTCCAGTCCAACTATGTACGGAACAATGCGAGCCTC                                   >  1:186375/1‑301`  
                                                                                                                                                                                                                                                                                        `CATGAACGAAAGACACTTAACAGGTGCGATTGTCCCCAATGGTAAACAGAAGGAAATGATTGTCCAGTTCCTGACACACG‑‑GTATGGAAGGATTGATGACATTCGTAGTACGTACATCATTTCGTGAGGCCATTAAGGACATGCACGAAGAGTATGCAGATAAGGACTCTTTCAAACAATCTCCTGCAACAGTACGGGAGGTGTTCTGATGTCTGACTACCTGAAAGTGCTGCAAGCAATCAAAAGTTGCCCTAAGACTTTCCAGTCCAACTATGTACGGAACGATGCGAGCCTCGTAGCGG                            <  1:431209/301‑1`  
                                                                                                                                                                                                                                                                                        `CATGAACGAAAGACACTTAACAGGTGCGATTGTCCCCAATGGTAAACAGAAGGAAATGATTGTCCAGTTCCTGACACACG‑‑GTATGGAAGGATTGATGACATTCGTAGTACGTACATCATTTCGTGAGGCCATTAAGGACATGCACGAAGAGTATGCAGATAAGGACTCTTTCAAACAATCTCCTGCAACAGTACGGGAGGTGTTCTGATGTCTGACTACCTGAAAGTGCTGCAAGCAATCAAAAGTTGCCCTAAGACTTTCCAGTCCAACTATGTACGGAACAATGCGAGCCTCGTAGCGG                            <  1:212560/301‑1`  
                                                                                                                                                                                                                                                                                             `ACGAAAGACACTTAACAGGTGCGATTGTCCCCAATGGTAAACAGAAGGAAATGATTGTCCAGTTCCTGACACACG‑‑GTATGGAAGGATTGATGACATTCGTAGTACGTACATCATTTCGTGAGGCCATTAAGGACATGCACGAAGAGTATGCAGATAAGGACTCTTTCAAACAATCTCCTGCAACAGTACGGGAGGTGTTCTGATGTCTGACTACCTGAAAGTGCTGCAAGCAATCAAAAGTTGCCCTAAGACTTTCCAGTCCAACTATGTACGGAACAATGCGAGCCTCGTAGCGGAGGCC                       <  1:80773/301‑1`  
                                                                                                                                                                                                                                                                                                    `ACACGTAACAGGTGCGATTGTCCCCAATGGTAAACAGAAGGAAATGATTGTCCAGTTCCTGACACACG‑‑GTATGGAAGGATTGATGACATTCGTAGTACGTACATCATTTCGTGAGGCCATTAAGGACATGCACGAAGAGTATGCAGATAAGGACTCTTTCAAACAATCTCCTGCAACAGTACGGGAGGTGTTCTGATGTCTGACTACCTGAAAGTGCTGCAAGCAATCAAAAGTTGCACTAAGACTTTCCAGTCCAACTATGTACGGAACAATGTGAGACTCGTAGCGGAGGCCGCTTCCC                >  1:540242/1‑301`  
                                                                                                                                                                                                                                                                                                     `acagTAACAGGTGCGATTGTCCCCAATGGTAAACAGAAGGAAATGATTGTCCAGTTCCTGACACACG‑‑GTATGGAAGGATTGATGACATTCGTAGTACGTACATCATTTCGTGAGGCCATTAAGGACATGCACGAAGAGTATGCAGATAAGGACTCTTTCAAACAATCTCCTGCAACAGTACGGGAGGTGTTCTGATGTCTGACTACCTGAAAGTGCTGCAAGCAATCAAAAGTTGCCCTAAGACTTTCCAGTCCAACTATGTACGGAACAATGCGAGCCTCGTAGCGGAGGCCGCTTCCCG               <  1:489663/297‑1`  
                                                                                                                                                                                                                                                                                                         `TAACAGGTGCGATTGTCCCCAATGGTAAACAGAAGGAAATGATTGTCCAGTTCCTGACACACG‑‑GTATGGAAGGATTGATGACATTCGTAGTACGTACATCATTTCGTGAGGACATTAAGGACATGCACGAAGAGTATGCAGATAAGGACTCTTTCAAACAATCTCCTGCAACAGTACGGGAGGTGTTCTGATGTCTGACTACCTGAAAGTGCTGCAAGCAATCAAAAGTTGCCCTAAGACTTTCCAGTCCAACTATGTACGGAACAATGCGAGCCTCGTAGCGGAGGCCGCTTCCCGTGGT           <  1:8808/301‑1`  
                                                                                                                                                                                                                                                                                                               `GTGCGATTGTCCCCAATGGTAAACAGAAGGAAATGATTGTCCAGTTCCTGACACACG‑‑GTATGGAAGGATTGATGACATTCGTAGTACGTACATCATTTCGTGAGGCCATTAAGGACATGCACGAAGAGTATGCAGATAAGGACTCTTTCAAACAATCTCCTGCAACAGTACGGGAGGTGTTCTGATGTCTGACTACCTGAAAGTGCTGCAAGCAATCAAAAGTTGCCCTAAGACTTTCCAGTCCAACTATGTACGGAACAATGCGAGCCTCGTAGCGGAGGCCGCTTCCCGTGGTCACATC     <  1:75108/301‑1`  
                                                                                                                                                                                                                                                                                                                  `CGATTGTCCCCAATGGTAAACAGAAGGAAATGATTGTCCAGTTCCTGACACACG‑‑GTATGGAAGGATTGATGACATTCGTAGTACGTACATCATTTCGTGAGGCCATTAAGGACATGCACGAAGAGTATGCAGATAAGGACTCTTTCATACAATCTCCTGCAACAGTACGGGAGGTGTTCTGATGTCTGACTACCTGAAAGTGCTGCAAGCAATCAAAAGTTTCCCTCAGACTTTCCAGTCCAACTATGTACGGAACAATTCGAGCCTCGTAGCGGAGGCCGCTTCCCTTGGTCACATCTCt  >  1:528533/1‑300`  

                                                                                                                                                                                                                                                                                                                                                                                                                                                                                                                                                                                                                                   
`CTGGTGCTCTCATCTGCAAACTGTGGATTATCAAGACCGAAGAGATGCTCGTAGAGAAAGGCTTGAAGCATGGCTGGGATGGGGACTTTGCGTACATGGCATGGGTACATGATGAAATCCAAGTAGGCTGCCGTACCGAAGAGATTGCTCAGGTGGTCATTGAGACCGCACAAGAAGCGATGCGCTGGGTTGGAGACCACTGGAACTTCCGGTGTCTTCTGGATACCGAAGGTAAGATGGGTCCTAATTGGGCGATTTGCCACTGATACAGGAGGCTACTCATGAACGAAAGACACTTAACAGGTGC‑‑‑‑‑‑‑‑‑‑‑‑‑‑‑‑‑‑‑‑‑‑‑‑‑‑‑‑‑‑‑‑‑‑‑‑‑‑‑‑‑‑‑‑‑‑‑‑‑‑‑‑‑‑‑‑‑‑‑‑‑‑‑‑‑‑‑‑‑‑‑‑‑‑‑‑‑‑‑‑‑‑‑‑‑‑‑‑‑‑‑‑‑‑‑‑‑‑‑‑‑‑‑‑‑‑‑‑‑‑‑‑‑‑‑‑‑‑‑‑‑‑‑‑‑‑‑‑‑‑‑‑‑‑‑‑‑‑‑‑‑‑‑‑‑‑‑‑‑‑‑‑‑‑‑‑‑‑‑‑‑‑‑‑‑‑‑‑‑‑‑‑‑‑‑‑‑‑‑‑‑‑‑‑‑‑‑‑‑‑‑‑‑‑‑‑‑‑‑‑‑‑‑‑‑‑‑‑‑‑‑‑‑‑‑‑‑‑‑‑‑‑‑‑‑‑‑‑‑‑‑‑‑‑‑‑‑‑‑‑‑‑‑‑‑‑‑‑‑‑‑‑‑‑‑‑‑‑‑‑‑‑‑‑‑‑‑‑‑‑‑‑‑‑‑‑‑‑‑‑‑‑‑‑‑‑‑‑‑‑‑‑‑‑‑‑‑‑‑‑‑‑  >  NC_001604.1/16202‑16508`  
`‑‑‑‑‑‑‑‑‑‑‑‑‑‑‑‑‑‑‑‑‑‑‑‑‑‑‑‑‑‑‑‑‑‑‑‑‑‑‑‑‑‑‑‑‑‑‑‑‑‑‑‑‑‑‑‑‑‑‑‑‑‑‑‑‑‑‑‑‑‑‑‑‑‑‑‑‑‑‑‑‑‑‑‑‑‑‑‑‑‑‑‑‑‑‑‑‑‑‑‑‑‑‑‑‑‑‑‑‑‑‑‑‑‑‑‑‑‑‑‑‑‑‑‑‑‑‑‑‑‑‑‑‑‑‑‑‑‑‑‑‑‑‑‑‑‑‑‑‑‑‑‑‑‑‑‑‑‑‑‑‑‑‑‑‑‑‑‑‑‑‑‑‑‑‑‑‑‑‑‑‑‑‑‑‑‑‑‑‑‑‑‑‑‑‑‑‑‑‑‑‑‑‑‑‑‑‑‑‑‑‑‑‑‑‑‑‑‑‑‑‑‑‑‑‑‑‑‑‑‑‑‑‑‑‑‑‑‑‑‑‑‑‑‑‑‑‑‑‑‑‑‑‑‑‑‑‑‑‑‑‑‑‑‑‑‑‑‑‑‑‑‑‑‑‑‑‑‑‑‑‑‑‑‑‑‑‑‑‑‑‑‑‑‑‑‑‑‑‑‑caggtgcGATTGTCCCCAATGGTAAACAGAAGGAAATGATTGTCCAGTTCCTGACACACG‑‑GTATGGAAGGATTGATGACATTCGTAGTACGTACATCATTTCGTGAGGCCATTAAGGACATGCACGAAGAGTATGCAGATAAGGACTCTTTCAAACAATCTCCTGCAACAGTACGGGAGGTGTTCTGATGTCTGACTACCTGAAAGTGCTGCAAGCAATCAAAAGTTGCCCTAAGACTTTCCAGTCCAACTATGTACGGAACAATGCGAGCCTCGTAGCGGAGGCCGCTTCCCGTGGTCACATCTCG  >  NC_001604.1/16970‑17269`

  

Alignment Legend

Aligned base mismatch/match (shaded by quality score): `ATCG/ATCG` `< 3 ≤` `ATCG/ATCG` `< 10 ≤` `ATCG/ATCG` `< 22 ≤` `ATCG/ATCG` `< 34 ≤` `ATCG/ATCG` `< 38 ≤` `ATCG/ATCG`

Unaligned base: `atcg`    Masked matching base: `atcg`    Alignment gap: `‑`    Deleted base: `‑`

Reads not counted as support for junction

read\_name Not counted due to insufficient overlap past the breakpoint.

read\_name Not counted due to not crossing MOB target site duplication.
